# COVID-19 Outcomes in Lung Transplant Recipients Following Pre-Exposure Prophylaxis With Tixagevimab-Cilgavimab During the Omicron BA.5 Surge: A Single Center Analysis

**DOI:** 10.3389/ti.2024.12061

**Published:** 2024-01-24

**Authors:** Saartje Demolder, Veronique Schaevers, Katrien Lagrou, Paul De Munter, Hanne Beeckmans, Geert M. Verleden, Laurent Godinas, Lieven J. Dupont, Pascal Van Bleyenbergh, Natalie Lorent, Robin Vos

**Affiliations:** ^1^ Department of Respiratory Diseases, University Hospitals Leuven, Leuven, Belgium; ^2^ Lung Transplant Team, University Hospitals Leuven, Leuven, Belgium; ^3^ Department of Laboratory Medicine and National Reference Center for Mycosis, University Hospitals Leuven, Leuven, Belgium; ^4^ Department of Microbiology, Immunology and Transplantation, KU Leuven, Leuven, Belgium; ^5^ Department of General Internal Medicine, University Hospitals Leuven, Leuven, Belgium; ^6^ Department of Chronic Diseases, Metabolism and Ageing (CHROMETA), Laboratory of Respiratory Diseases and Thoracic Surgery (BREATHE), KU Leuven, Leuven, Belgium

**Keywords:** COVID-19, lung transplantation, pre-exposure prophylaxis, tixagevimab-cilgavimab, outcome predictors

## Abstract

Lung transplant (LTx) recipients are at high risk for COVID-19 related morbidity and mortality. Data regarding pre-exposure prophylaxis (PrEP) with tixagevimab-cilgavimab in this population are scarce. We therefore evaluated COVID-19 breakthrough infections and COVID-19 related complications after PrEP in a retrospective single-center study, including 264 LTx recipients who received PrEP between June 2022 and December 2022, when Omicron BA.5 was the dominant circulating SARS-CoV-2 variant. PrEP was indicated for fully vaccinated patients with poor seroconversion (anti-S <260 BAU/mL). COVID-19 breakthrough infection after PrEP occurred in 11.0% within the first 3 months, increasing to 17.4% within 6 months. Hospitalization rate rose from 27.6% to 52.9% (*p* = 0.046), while ICU admissions and COVID-19 mortality remained low, respectively occurring in 6.5% and 4.3% of patients with breakthrough infection within 6 months. COVID-19 breakthrough infection and associated hospitalization remained an important problem during the Omicron BA.5 surge in fully vaccinated LTx recipients with deficient seroconversion, despite PrEP with tixagevimab-cilgavimab. However, ICU admissions and COVID-19 mortality were low. Waning of neutralizing effects of PrEP and changing circulating SARS-CoV-2 variants may explain increases in COVID-19 infections and hospitalizations over time after PrEP, highlighting the need for novel, long-term effective PrEP strategies in these high-risk patients.

## Graphical Abstract

**Figure:**
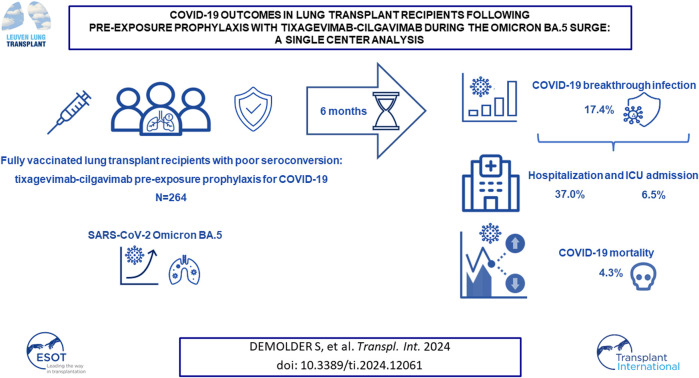

## Introduction

The incidence of COVID-19 infections in solid organ transplant (SOT) recipients is high in the Omicron era, especially in lung transplant (LTx) recipients, which are particularly at risk for COVID-19 related complications (i.e., hospitalization, severe disease, intensive care unit (ICU)-admission, respiratory failure, and death) [1–6] due to suboptimal or ineffective antibody responses following prior vaccination [7, 8]. As we previously reported, LTx recipients in our center demonstrated poor antibody seroconversion rates of only 47% after the third (“booster”) vaccine dose, and the lowest antibody titers compared with other SOT recipients, resulting in the highest rates of severe breakthrough infection (10.5%) and death (2.5%) [9]. These poorer outcomes in the LTx population highlight the importance of LTx-specific studies and further research in LTx regarding effective prevention and treatment options.

Tixagevimab-cilgavimab (Evusheld^®^) is a long-acting dual monoclonal antibody against the SARS-CoV-2 spike protein, which has been available for pre-exposure prophylaxis (PrEP)—in adjunction to vaccination - in severely immunocompromised patients in Belgium since May 2022, as it retained activity against some circulating SARS-CoV-2 Omicron variants. Emerging international evidence of prophylactic treatment with tixagevimab-cilgavimab suggest efficacy for COVID-19 related complications in SOT recipients, but data in LTx recipients are scarce [10–16].

## Patients and Methods

### Study Population

We conducted a retrospective single-center study of lung transplant recipients receiving tixagevimab-cilgavimab PrEP during the Omicron period. We included all consecutive LTx recipients who received tixagevimab-cilgavimab PrEP in our institution between 10th June 2022 and 13th December 2022. As per national recommendations [17], the indication for tixagevimab-cilgavimab PrEP in immunocompromised patients was: SARS-CoV-2 anti-Spike antibody titers <260 Binding Antibody Units (BAU)/mL (AdviseDx SARS-CoV-2 IgG II assay, Abbott, IL, United States) assessed >14 days after the second COVID-19 booster vaccine (i.e., fully vaccinated patients with insufficient seroconversion, considered at risk for severe COVID-19). Tixagevimab-cilgavimab was provided by the National Health Authorities to eligible immunocompromised patients, in whom this antibody titer cut-off was mandatory and thus country-specific. PrEP was administered as a single dose (tixagevimab 150 mg/cilgavimab 150 mg, two separate consecutive intramuscular injections). The dominant circulating SARS-CoV-2 variant in Belgium during the study period was Omicron BA.5. In our center, PrEP was routinely offered to all patients with the above indication by their treating transplant physician at their outpatient follow-up visits, provided they had no symptoms suggestive of COVID-19, and subsequently administered upon informed consent. Patients were subsequently monitored for 1 h after PrEP for possible serious adverse events, which required reporting to the National Health Authorities in case these occurred.

### Data Collection

Data on demographics, reports of positive SARS-CoV-2 PCR, and clinical outcomes of interest were extracted from the patients’ electronic medical records. Demographics included age, sex, body mass index (BMI), time and type of transplant, type of immunosuppressive regimen and comorbidities, including diabetes mellitus, chronic kidney disease and heart disease. Pre-existing chronic kidney disease was defined as severe chronic renal insufficiency stage 4, with eGFR <30 mL/min/1.73 m^2^. Pre-existing heart disease was defined as ischemic heart disease (IHD), non-ischemic heart failure with or without reduced ejection fraction (HF), or arrythmia (AR).

SARS-CoV-2 positivity was defined by a positive PCR test; rapid antigen tests were not included, due to possible reporting bias. All patients with new clinical symptoms suggestive for COVID-19 were instructed to undergo COVID-19 PCR testing, either by their general practitioner or at the transplant center, which allowed to clinically assess the patient’s symptoms at each new COVID-19 diagnosis, and refer/admit to hospitalization, if deemed necessary. The PCR results were prospectively documented in the patient’s medical records and a centralized database.

Prevalence of symptomatic COVID-19 breakthrough infections, hospitalization, ICU admission and all-cause mortality were assessed up to 6 months after tixagevimab-cilgavimab administration. Patients with a recently confirmed COVID-19 infection prior to (<3 weeks) (*n* = 6) or post (<5 days) PrEP (*n* = 0) were excluded for analysis. We allocated the term *mild* disease to patients who solely required ambulatory care, *moderate* disease to those who were hospitalized and *severe* disease to those in need of intensive care unit management.

### Statistical Analysis

Patient characteristics and variables of interest/endpoints were summarized using descriptive statistics, and results are expressed as total value, proportions, mean (standard deviation) or median (interquartile range), wherever appropriate. Proportions were compared using Chi-square testing. Groups were compared using paired t-tests, unpaired Mann-Whitney tests, or Wilcoxon matched-pairs signed rank tests for repeated measures. Correlation analyses were performed using Spearman rank testing. A *p*-value <0.05 was considered significant. All statistical analyses were performed using GraphPad Prism 10.0 (Dotmatics, Boston, MA, United States).

### Ethics Approval and Consent to Participate

At listing for LTx, all patients provide signed informed consent to use their clinical data for scientific research purposes by affiliated researchers of University Hospitals Leuven. Tixagevimab-cilgavimab PrEP was standard of care and administered after oral consent of the LTx recipient following therapeutic proposal by their treating physician. The institutional Ethics Review Board waived approval for the current retrospective, observational study (MP024291, S68119).

## Results

### Study Population

**FIGURE 1:**
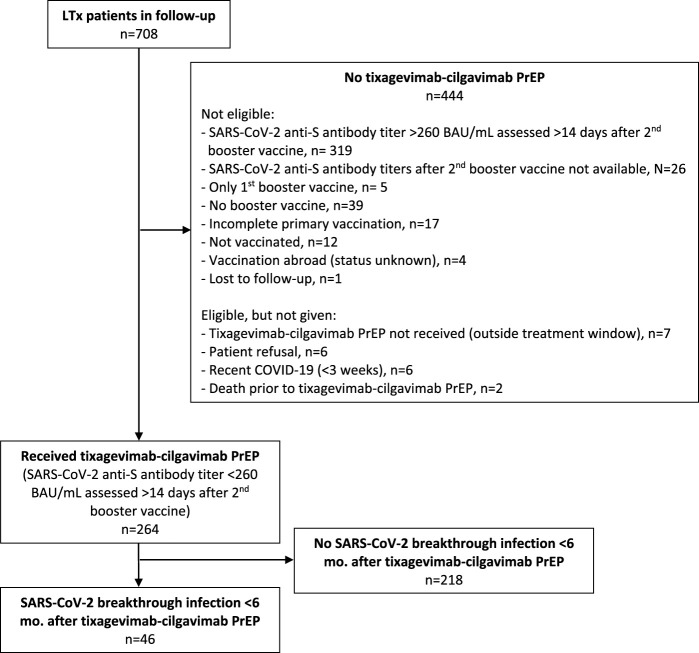
Flow chart. Legend: Abbreviations: BAU, Binding Antibody Unit; LTx, lung transplantation; PrEP, pre-exposure prophylaxis.

**FIGURE 2:**
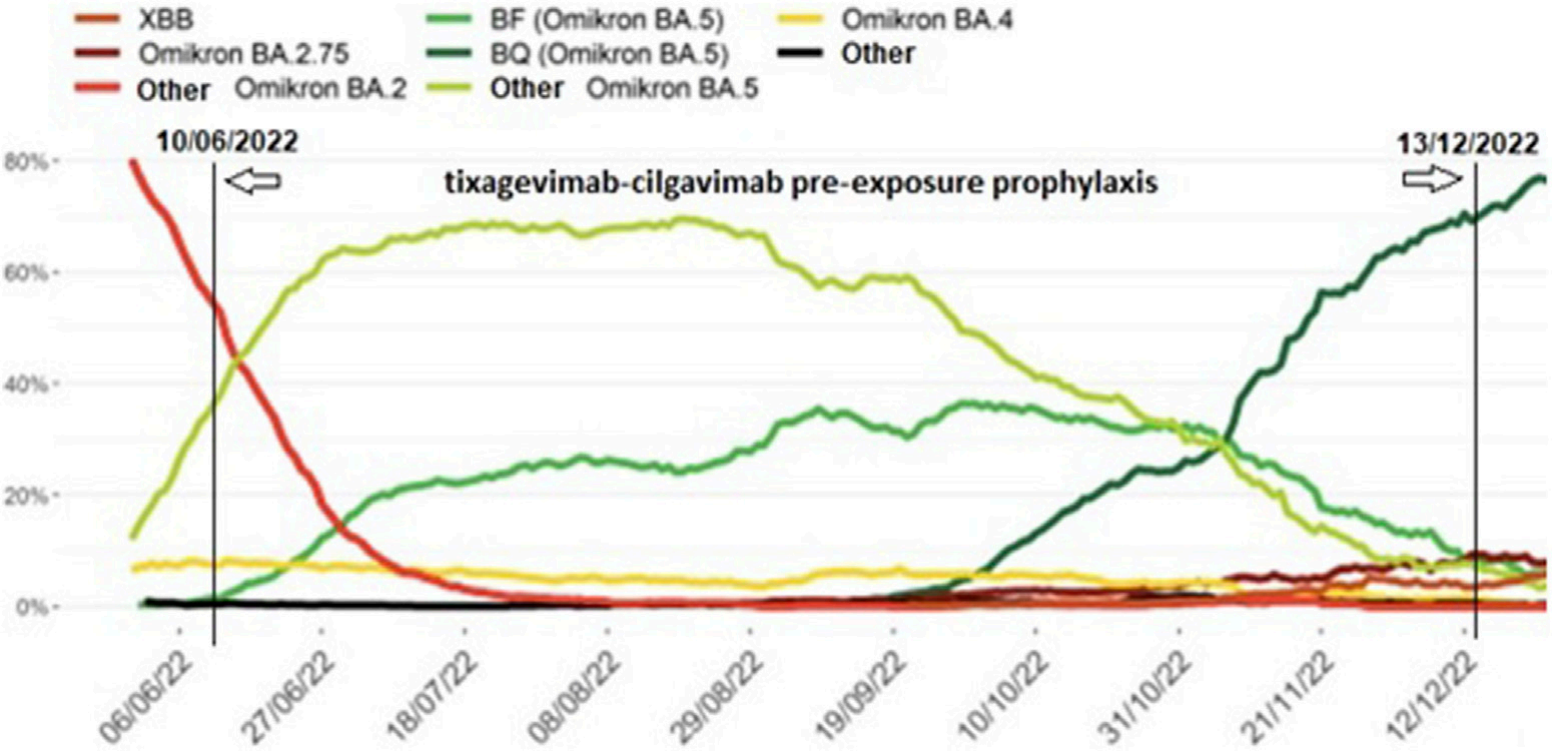
Prevalence of SARS-CoV-2 Omicron subvariants identified during baseline surveillance in Belgium from June 2022 until January 2023, 7-day moving average. Legend: Different SARS-CoV-2 Omicron subvariants (coloured lines) during the tixagevimab-cilgavimab pre-exposure prophylaxis administration period in our patient cohort (10th June 2022 to 13th December 2022, vertical lines) (adapted from [18]).

**FIGURE 3:**
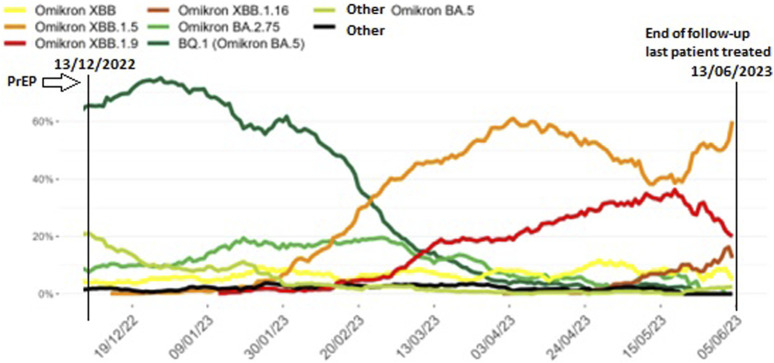
Prevalence of SARS-CoV-2 Omicron subvariants identified during baseline surveillance in Belgium from November 2022 until June 2023, 7-day moving average. Legend: Different SARS-CoV-2 Omicron subvariants (coloured lines) in the 6 months after the tixagevimab-cilgavimab pre-exposure prophylaxis (PrEP) administration period in our patient cohort (13th December 2022 to 13th June 2023, vertical lines) (adapted from [19]).

A total of 285 LTx recipients (40.3% of our total LTx population of 708 patients) were eligible for tixagevimab-cilgavimab PrEP. The flow chart of included and excluded patients is given in Figure 1. The 264 included patients received PrEP between 10th June 2022 and 13th December 2022, at which moment Omicron BQ.1 (a subvariant of BA.5) replaced BA.5 (BF) as the main circulating Omicron variant in Belgium (Figures 2, 3), and consequently administration of tixagevimab-cilgavimab PrEP was no longer recommended by the National Health Authorities because of reduced neutralizing efficacy against this BQ.1 subvariant [19, 20]. Median time between LTx and PrEP was 79.8 (43.0–138.5) months, and time between last SARS-CoV-2 booster vaccine and PrEP 173 (145–202.5) days. No serious adverse events (i.e., serious hypersensitivity reactions, including anaphylaxis) were seen immediately following PrEP administration, in addition, none of the included patients experienced a cardiovascular serious adverse event (i.e., myocardial infarction or stroke) during the 6-month post-PrEP study period.

Patient demographics are summarized in Table 1. Most patients were female (51.5%), and median age was 64 (55–68) years. 254 patients (96.2%) underwent lung transplantation only, 5 patients combined heart-lung transplantation, 3 others combined liver-lung transplantation, 1 patient a kidney-lung transplantation and another a liver-kidney-lung transplantation. Most patients (*n* = 235, 89.0%) were on a tacrolimus-based immunosuppressive regimen. None of the included patients was treated with a m-TOR inhibitor, had nor had received lymphocyte-depleting treatment (e.g., total lymphoid irradiation, antithymocyte globulin, or rituximab) for progressive chronic lung allograft dysfunction (CLAD) within 4–6 weeks prior to PrEP administration (as patients were required to be clinically stable to safely allow PrEP administration). Median BMI in our study population was in the normal range (23.4 (20.7–26.8), chronic lung allograft dysfunction (CLAD) was present in 27.7% (*N* = 73) of patients, 30.7% suffered from diabetes mellitus (*n* = 81), 17.8% from chronic renal insufficiency (*n* = 47) and 26.5% from pre-existing heart disease (*n* = 70).

**TABLE 1:** Demographics of all study patients.

Demographic variable	All study patients (*n* = 264)
Age, y, median (IQR)	64 (55–68)
Male sex, *n* (%)	128 (48.5%)
Female sex, *n* (%)	136 (51.5%)
Single organ (lung only) transplant, *n* (%)	254 (96.2%)
Heart-lung transplant, *n* (%)	5 (1.9%)
Liver-lung transplant, *n* (%)	3 (1.1%)
Kidney-lung transplant, *n* (%)	1 (0.4%)
Liver-kidney-lung transplant, *n* (%)	1 (0.4%)
Tacrolimus-based immunosuppressant regimen, *n* (%)	235 (89.0%)
Cyclosporine A-based immunosuppressant regimen, *n* (%)	29 (11.0%)
Chronic lung allograft dysfunction, *n* (%)	73 (27.7%)
Bronchiolitis Obliterans Syndrome	55 (75.3%)
Mixed	6 (8.2%)
Restrictive Allograft Syndrome	12 (16.4%)
BMI, median (IQR)	23.4 (20.7–26.8)
Diabetes mellitus, *n* (%)	81 (30.7%)
Chronic renal insufficiency (CKD stage 4), *n* (%)	47 (17.8%)
Heart disease, *n* (%)	70 (26.5%)
Ischemic heart disease	15 (5.7%)
Non-ischemic heart failure with or without preserved ejection fraction	13 (4.9%)
Arrythmia	42 (15.9%)
Months between LTx and PrEP, median (IQR)	79.8 (43.0–138.5)
SARS-CoV-2 anti-Spike antibody titers prior to PrEP, BAU/mL (IQR)	13.2 (3.0–91.3)

Abbreviations: BAU, binding antibody unit; BMI, body mass index; CKD, chronic kidney disease; LTx, lung transplantation; PrEP, pre-exposure prophylaxis.

Median SARS-CoV-2 anti-Spike antibody titer prior to PrEP was 13.2 (3.0–91.3) BAU/mL in the 264 included patents (i.e., <260 BAU/mL to be eligible for PrEP). In comparison, median SARS-CoV-2 anti-Spike antibody titer was 2243.0 (716.3–4785.0) BAU/mL in the 319 patients not eligible for PrEP (*p* < 0.0001).

### COVID-19 Outcomes at 3 Months Follow-Up

A total of 29 (11.0%) patients had confirmed COVID-19 within 3 months after PrEP, of whom 8 (27.6%) were hospitalized, one of whom was admitted to ICU (3.4%). There was one COVID-19 related death (3.4%), being the ICU-hospitalized patient. Median time between PrEP and breakthrough infection was 43 (28–50) days.

Relevant clinical variables and immunosuppressant regimen in the patients with or without breakthrough infection within 3 months after PrEP are summarized in Table 2; and did not differ between both groups. All, but one, COVID-19 patients were single organ (lung only) transplant recipients. There was one COVID-19 patient who had received a combined heart-lung transplantation, this patient only suffered from mild disease (i.e., not hospitalized).

**TABLE 2:** Clinical variables in the lung transplant recipients with or without COVID-19 within 3 months after tixagevimab-cilgavimab PrEP.

Clinical variable	No COVID-19 (*n* = 235)	COVID-19 infected (*n* = 29)	*p*-value
Age, median (IQR)	64 (55–68)	65 (56–68)	0.87
Gender, Male/Female, *n*	114/121 (48.5/51.5%)	14/15 (48.3/51.7%)	0.98
BMI, median (IQR)	23.2 (20.5–26.6)	24.1 (21.4–29.2)	0.21
Diabetes mellitus *n* (% of total)	74 (31.5%)	7 (24.1%)	0.42
Chronic renal insufficiency, *n* (% of total)	39 (16.6%)	8 (27.6%)	0.14
Heart disease, *n* (% of total)	59 (25.1)	11 (37.9%)	0.14
Ischemic Heart Disease	15 (6.4%)	0 (0.0%)	
Heart Failure	11 (4.7%)	2 (6.9%)	
Arrythmia	33 (14.0%)	9 (31.0%)	
Chronic lung allograft dysfunction, *n* (% of total)	62 (26.4%)	11 (37.9%)	0.19
Bronchiolitis Obliterans Syndrome	47 (19.1%)	8 (27.6%)	
Mixed	5 (2.1%)	1 (3.4%)	
Restrictive Allograft Syndrome	10 (4.2%)	2 (6.9%)	
Immunosuppressant regimen, *n* (% of total)			0.82
TAC/MMF/CS	131 (55.7%)	13 (44.8%)	
TAC/AZA/CS	45 (19.1%)	6 (20.7%)	
CSA/MMF/CS	17 (7.2%)	2 (6.9%)	
CSA/AZA/CS	4 (1.7%)	1 (3.4%)	
TAC/CS	32 (13.6%)	7 (24.1%)	
TAC/AZA	1 (0.4%)	0 (0.0%)	
CSA/CS, *n* (%)	5 (2.1%)	0 (0.0%)	
Months of follow-up since transplant, median (IQR)	79.9 (44.3–138.2)	78.6 (34.3–152.8)	0.82
Days between transplant and PrEP, median (IQR)	2,214 (1,051–3,926)	2,095 (743–4,348)	0.83
Days between last booster vaccine and PrEP, median (IQR)	181.5 (166.0–200.5)	173.0 (143.5–202.5)	0.33

Abbreviations: AZA, azathioprine; BMI, body mass index; COVID-19, Coronavirus Disease 2019; CS, corticosteroids; CSA, cyclosporine A; MMF, mycophenolate mofetil; PReP, pre-exposure prophylaxis; TAC,tacrolimus.

### COVID-19 Outcomes at 6 Months Follow-Up

**FIGURE 4:**
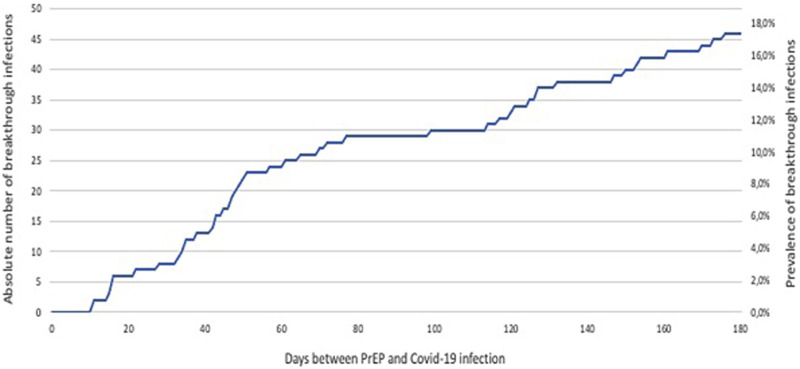
Evolution of COVID-19 breakthrough infections in lung transplant recipients during 6 months follow-up after tixagevimab-cilgavimab PrEP. Legend: Abbreviations: PrEP, pre-exposure prophylaxis.

Another 17 patients had confirmed COVID-19 within 3–6 months after PrEP, resulting in a total of number of 46 patients (17.4% of all PrEP patients) with COVID-19 breakthrough infection within 6 months after PrEP (Figure 4). Demographics of all lung transplant recipients with COVID-19 within 6 months after tixagevimab-cilgavimab PrEP, according to time to COVID-19 breakthrough infection since PrEP (i.e., <3 months and 3–6 months), are summarized in Table 3. There were no significant differences between patients infected <3 months vs. 3–6 months after PrEP.

**TABLE 3:** Characteristics of lung transplant recipients with COVID-19 within 6 months after tixagevimab-cilgavimab PrEP.

Nr	Days between PrEP and COVID-19 positive	Sex	Age (y)	BMI	DM	CKD	HD	CLAD (type)	Lymphoid-depleting treatment pre-PrEP (type)	Days between LTx and booster vaccine	Days between LTx and PrEP	Days between booster and PrEP	Anti-S Abs pre-PrEP (BAU/mL)	Hospitalization (duration, days)	ICU	All-cause mortality	COVID-19 mortality	High flow nasal oxygen	MV or NIV	Dialysis	CAPA	Remdesivir	Nirmatrelvir/Ritonavir
<3 Months																							
1	11	M	65	28.1	No	No	Yes (AR)	No	No	942	1,117	175	3.0	Yes (3)	No	No	No	No	No	No	No	No	No
2	11	F	52	31.3	No	No	No	**Yes (BOS)**	No	3,647	3,850	203	223.3	No	No	No	No	No	No	No	No	No	No
3	15	F	68	27.5	No	**Yes**	No	No	No	1,368	1,421	53	8.9	No	No	No	No	No	No	No	No	No	No
4	16	F	58	31.2	No	No	No	**Yes (RAS)**	No	1,929	2,095	166	24.9	No	No	No	No	No	No	No	No	No	No
5	16	F	63	15.8	No	**Yes**	No	No	No	4,196	4,383	187	3.0	No	No	No	No	No	No	No	No	No	No
6	16	F	75	24.7	No	No	No	No	No	3,175	3,395	220	110.1	No	No	No	No	No	No	No	No	No	No
7	22	F	66	34.2	No	No	No	No	No	566	732	166	3.0	No	No	No	No	No	No	No	No	No	No
8	28	F	62	22.0	No	**Yes**	No	No	No	594	761	167	3.0	No	No	No	No	No	No	No	No	No	No
9	33	M	57	21.4	No	No	No	**Yes (RAS)**	No	2,600	2,830	230	3.0	**Yes (40)**	**Yes**	**Yes**	**Yes**	**Yes**	No	No	No	**Yes**	No
10	34	M	69	28.1	No	No	**Yes (AR)**	**Yes (BOS)**	No	1,848	2,036	188	170.5	No	No	No	No	No	No	No	No	No	No
11	35	F	65	21.4	No	**Yes**	No	No	No	614	736	122	3.0	No	No	No	No	No	No	No	No	No	No
12	35	F	45	21.8	**Yes**	No	**Yes (AR)**	No	No	6,587	6,768	181	3.0	No	No	No	No	No	No	No	No	No	No
13	38	M	71	19.6	No	**Yes**	**Yes (HF, AR)**	No	No	5,589	5,767	178	3.0	No	No	No	No	No	No	No	No	No	No
14	42	F	45	24.0	**Yes**	**Yes**	No	**Yes (BOS)**	No	7,366	7,553	187	3.0	**Yes (11)**	No	No	No	No	No	No	**Yes**	No	No
15	43	M	66	29.7	No	**Yes**	No	No	No	655	840	185	3.0	No	No	No	No	No	No	No	No	No	No
16	43	M	51	24.5	**Yes**	No	No	No	No	7,672	7,865	193	171.6	No	No	No	No	No	No	No	No	No	No
17	45	M	74	23.4	No	No	**yes (AR)**	No	No	4,182	4,313	131	3.0	No	No	No	No	No	No	No	No	No	No
18	47	M	57	30.7	No	No	No	No	No	543	732	189	3.0	No	No	No	No	No	No	No	No	No	No
19	47	M	69	23.8	No	No	No	No	No	4,216	4,493	277	52.6	**Yes (3)**	No	No	No	No	No	No	No	No	No
20	48	M	57	19.6	**Yes**	No	No	No	No	524	688	164	5.0	No	No	No	No	No	No	No	No	No	No
21	49	F	46	31.6	**Yes**	No	No	No	No	584	750	166	3.8	No	No	No	No	No	No	No	No	No	No
22	50	F	66	15.5	**Yes**	No	**Yes (AR)**	**No**	No	−97	259	356	177.9	No	No	No	No	No	No	No	No	No	No
23	51	F	67	17.1	No	No	No	**Yes (BOS)**	**Yes (TLI)**	uk	3,205	uk	3.0	No	No	No	No	No	No	No	No	No	No
24	57	F	55	35.5	No	No	No	No	No	512	670	158	235.6	No	No	No	No	No	No	No	No	No	No
25	61	M	39	20.2	No	No	No	No	No	295	364	69	3.0	**Yes (5)**	No	No	No	No	No	No	No	No	No
26	65	M	68	28.7	**Yes**	**Yes**	**Yes (AR)**	**Yes (Mixed)**	No	2,675	2,907	232	7.7	**Yes (11)**	No	No	No	No	No	No	No	No	No
27	70	F	68	23.3	No	No	No	No	No	813	995	182	3.0	No	No	No	No	No	No	No	No	No	No
28	72	M	67	24.1	No	No	**Yes (HF, AR)**	No	No	2,128	2,295	167	3.0	**Yes (6)**	No	No	No	No	No	No	No	No	No
29	77	M	72	25.1	No	No	**Yes (AR)**	**Yes (BOS)**	No	5,216	5,461	245	3.0	**Yes (3)**	No	No	No	No	No	No	No	No	No
3–6 Months																							
30	99	M	63	30.1	**Yes**	No	**Yes (HF)**	No	No	445	661	216	237.0	No	No	No	No	No	No	No	No	No	No
31	114	V	68	19.3	No	No	No	No	No	973	1,210	237	3.9	**Yes (7)**	No	No	No	No	No	No	No	**Yes**	No
32	117	M	66	27.0	No	No	No	No	No	621	735	114	240.7	No	No	No	No	No	No	No	No	No	No
33	120	V	71	28.1	**Yes**	No	**Yes (IHD)**	No	No	1,967	2,106	139	3.0	**Yes (10)**	No	No	No	No	No	No	No	No	No
34	121	V	69	15.8	No	No	No	**Yes (BOS)**	No	7,952	8,125	173	45.8	**Yes (21)**	No	No	No	No	No	No	No	No	No
35	125	M	67	22.3	**Yes**	No	**Yes (AR)**	No	No	883	1142	259	29.5	**Yes (10)**	No	No	No	No	No	No	No	**Yes**	No
36	127	M	54	25.6	No	No	**yes (AR)**	**yes (BOS)**	No	2,974	3,118	144	3.0	No	No	No	No	No	No	No	No	No	No
37	127	V	66	26.9	No	**Yes**	No	No	No	381	552	171	4.6	**Yes (4)**	No	No	No	No	No	No	No	No	No
38	132	V	50	18.8	No	**Yes**	**Yes (AR)**	No	No	4,378	4,512	134	3.0	**Yes (19)**	**Yes**	No	No	**Yes**	**Yes (NIV)**	No	No	No	No
39	147	M	68	23.0	No	No	**Yes (AR)**	No	No	3,324	3,516	192	3.0	No	No	No	No	No	No	No	No	No	No
40	150	M	64	26.4	No	No	**Yes (IHD)**	**Yes (BOS)**	No	633	804	171	3.1	**Yes (5)**	No	No	No	No	No	No	No	No	No
41	153	V	70	17.5	No	**Yes**	No	No	No	3,296	3,477	181	23.3	No	No	No	No	No	No	No	No	No	No
42	154	V	52	20.0	**Yes**	No	No	No	No	3,629	3,707	78	3.0	No	No	No	No	No	No	No	No	No	No
43	161	V	44	15.8	No	No	No	No	No	103	268	165	95.0	**Yes (18)**	No	No	No	No	No	No	**Yes**	**Yes**	No
44	170	M	73	24.0	No	**Yes**	No	No	No	2,695	2,826	131	88.4	**Yes (32)**	**Yes**	**Yes**	**Yes**	**Yes**	**Yes (MV)**	No	No	No	No
45	173	M	62	34.4	**Yes**	No	No	No	No	548	738	190	3.0	No	No	No	No	No	No	No	No	No	No
46	176	V	61	24.6	No	No	No	No	No	403	557	154	48.8	No	No	No	No	No	No	No	No	No	No

Overview of the lung transplant recipients with COVID-19 within 6 months after tixagevimab-cilgavimab PrEP, according to time to COVID-19 breakthrough infection since PrEP (i.e., <3 months and 3–6 months). Abbreviations: AR, arrythmia; BMI, body mass index; BOS, bronchiolitis obliterans syndrome; CAPA, COVID-19 associated pulmonary aspergillosis; CLAD, chronic lung allograft dysfunction; CKD, chronic kidney disease defined as pre -existing chronic renal insufficiency stage 4, with eGFR <30 mL/; DM, diabetes mellitus; HD, pre-existing heart disease; HF, non-ischemic heart failure with or without preserved ejection fraction; ICU, intensive care unit; IHD, ischemic heart disease; LTx, lung transplantation; MV, mechanical ventilation; NIV, non-invasive ventilation; PrEP, pre-exposure prophylaxis; RAS, restrictive allograft syndrome; TLI, total lymphoid irradiation; UK, unknown.

Bold values visually represent the respctive parameter being present (YES), rather than absent (NO).

Main outcomes in the 46 COVID-19 patients, according to time of breakthrough infection after PrEP, are summarized in Table 4. Median time to breakthrough infection was 54 (36–124) days.

**TABLE 4:** Main outcomes of lung transplant recipients with COVID-19 breakthrough infection, according to time of follow-up after tixagevimab-cilgavimab PrEP.

Patients with COVID*-*19 after PrEP	0–6 months follow-up (*n* = 46)	0–3 months follow-up (*n* = 29)	3–6 months follow-up (*n* = 17)	*p*-value (0–3 vs. 3–6 months)
Hospitalization, *n* (%)	17 (37.0%)	8 (27.6%)	9 (52.9%)	0.046
ICU admission, *n* (%)	3 (6.5%)	1 (3.4%)	2 (11.8%)	0.14
COVID-19 related mortality, *n* (%)	2 (4.3%)	1 (3.4%)	1 (5.9%)	0.35

Abbreviations: ICU, intensive care unit; PrEP, pre-exposure prophylaxis.

Of these patients, 17 (37%) were hospitalized and 3 (6.5%) were admitted to ICU. Mortality in the COVID-19 group (4.3%) was comparable to the total study group (5.3%) (*p* = 0.37). In total 11 out of 46 COVID-19 patients had pre-existing CLAD (8 BOS, 1 Mixed, 2 RAS). Of these, 1 patient demonstrated CLAD (BOS) progression with a FEV_1_ decline of >10% during the 6 months study period, 1 patient (RAS) died due to COVID-19, and in the 9 other patients FEV_1_ remained stable pre-COVID-19 vs. 6 months post-COVID-19 [FEV_1_ 1.66 (1.18–2.15) L vs. 1.72 (1.05–1.96) L, *p* = 0.74].

Relevant clinical variables according to disease severity (mild vs. moderate to severe) in these 46 COVID-19 patients are summarized in Table 5. Patients presenting with mild disease were on average sooner infected after PrEP compared to patients requiring hospitalization: 47 (34–99) vs. 114 (61–121) days (*p* = 0.006). Patients with pre-existing heart disease tended to be more hospitalized (47.1% vs. 27.6%, *p* = 0.09), but no increased hospitalization risk was seen for concurrent CLAD, diabetes mellitus or chronic renal insufficiency, nor for type of immunosuppressive regimen.

**TABLE 5:** Characteristics according to disease severity in lung transplant recipients with COVID-19 breakthrough infection during 6 months follow-up after tixagevimab-cilgavimab PrEP.

	All COVID-19 infected patients (*n* = 46)	Mild COVID-19 (ambulatory) (*n* = 29)	Moderate to severe COVID-19 (hospitalization) (*n* = 17)
Age, y, mean (SD)	62 (9.0%)	62 (7.9%)	62 (10.8%)
Male sex, *n* (%)	22 (47.8%)	12 (41.0%)	10 (59.0%)
Female sex, *n* (%)	24 (52.2%)	17 (59.0%)	7 (41.0%)
Days between PrEP and infection, median (IQR)	54 (36–124)	47 (34–99)	114 (61–121)*
Single organ (lung) transplant, *n* (%)	45 (97.8%)	28 (96.6%)	17 (100.0%)
Heart-lung transplant, *n* (%)	1 (2.2%)	1 (3.4%)	0 (0.0%)
Liver-lung transplant, *n* (%)	0 (0.0%)	0 (0.0%)	0 (0.0%)
Kidney-lung transplant, *n* (%)	0 (0.0%)	0 (0.0%)	0 (0.0%)
Liver-kidney-lung transplant, *n* (%)	0 (0.0%)	0 (0.0%)	0 (0.0%)
Chronic lung allograft dysfunction, *n* (%)	11 (23.9%)	5 (17.2%)	6 (35.3%)
Diabetes mellitus, *n* (%)	12 (26.1%)	8 (27.6%)	4 (23.5%)
Chronic renal insufficiency (CKD stage 4), *n* (%)	12 (26.1%)	7 (24.1%)	5 (29.4%)
Heart disease, *n* (%)	16 (34.8%)	8 (27.6%)	8 (47.1%)
Tacrolimus-based immunosuppression, *n* (%)	40 (87.0%)	25 (86.2%)	15 (88.2%)
Cyclosporine A-based immunosuppression, *n* (%)	6 (13.0%)	4 (13.8%)	2 (11.8%)
Mycophenolate as cell cycle inhibitor, *n* (%)	26 (56.5%)	15 (51.7%)	11 (64.7%)
SARS-CoV-2 anti-Spike Antibody titers pre-PrEP, BAU/mL (IQR)	3.1 (3.0–49.8)	3.0 (3.0–140.3)	3.1 (3.0–37.7)
All-cause mortality, *n* (%)	3 (6.5%)	0 (0.0%)	3 (17.6%)**
COVID-19 related mortality, *n* (%)	2 (4.3%)	0 (0.0%)	2 (11.8%)

Abbreviations: BAU, binding antibody unit; PrEP, pre-exposure prophylaxis.

*p = 0.006 vs. mild COVID-19, **p = 0.0139 vs. mild COVID-19.

Furthermore, only one COVID-19 patient had received lymphoid-depleting treatment with total lymphoid irradiation for allograft rejection within the 6 months prior to PrEP, none had received antithymocyte globulin treatment. Only four COVID-19 patients received remdesivir, all were hospitalized, and one died in the ICU (Table 3). None of the infected patients received nirmatrelvir/ritonavir for COVID-19 treatment in our programme.

Notably, SARS-CoV-2 anti-Spike antibody titer pre-PrEP was lower in COVID-19 patients (*n* = 46) compared to non-infected patients (*n* = 218): 3.1 (3.0–49.8) vs. 18.5 (3.0–98.3) BAU/mL (*p* = 0.039). Likewise, in a subgroup of patients (*n* = 36, 13.6%) in whom SARS-CoV-2 anti-Spike antibody titer was measured post-PrEP, a significantly lower antibody titer was seen in COVID-19 patients (*n* = 10) compared to non-infected patients (*n* = 26): 1261.6 (857.7–1835.1) vs. 2201.7 (1380.4–3405.7) BAU/mL (*p* = 0.0185) (Table 6).

**TABLE 6:** SARS-CoV-2 anti-Spike antibody titers post-PrEP.

	All patients (*n* = 36)	Non-infected patients (*n* = 26)	COVID-19 infected patients (*n* = 10)
SARS-CoV-2 anti-Spike Antibody titers post-PrEP, BAU/mL (IQR)	1742.5 (1100.6–2934.1)	2201.7 (1380.4–3405.7)	1261.6 (857.7–1835.1)*
Time between PrEP and measurement of SARS-CoV-2 anti-Spike Antibody titers post-PrEP, days (IQR)	70.0 (30.8–96.0)	63.0 (25.5–94.0)	80.5 (46.3–104.8)

SARS-CoV-2 anti-Spike antibody titers post-PrEP were only available in 36/264 (13.6%) of included patients: in 10/46 patients (21.7%) with COVID-19 breakthrough infection and in 26/218 (11.9%) non-infected patients. *p = 0.0185 versus non-infected patients (time between PrEP and Antibody measurement was similar in both groups, *p* = 0.315). Abbreviations: BAU, binding antibody unit; PrEP, pre-exposure prophylaxis.

In the COVID-19 patients, anti-Spike antibody titer pre-PrEP was similar in patients infected <3 months compared to those infected after 3–6 months: 3.0 (3.0–27.2) vs. 4.6 (3.0–91.7) (*p* = 0.16). Also, anti-Spike antibody titer pre-PrEP was similar in patients with mild COVID-19 compared to those with moderate to severe COVID-19: 3.0 (3.0–140.3) vs. 3.1 (3.0–37.7) BAU/mL (*p* = 0.65) (Table 4).

Relevant COVID-19 related outcome variables in the 17 *hospitalized* COVID-19 patients are summarized in Table 7. Overall, hospitalization duration was short (mean 12.2 ± 10.7 days), and respiratory support requiring non-invasive ventilation (*n* = 1), intubation with mechanical ventilation (*n* = 1), or extracorporeal support (*n* = 1) was rarely needed. COVID-19 associated pulmonary aspergillosis was diagnosed in two patients.

**TABLE 7:** Characteristics of hospitalized lung transplant recipients with COVID-19 breakthrough infection, according to time of follow-up after tixagevimab-cilgavimab PrEP.

Patients with *moderate to severe* disease (hospitalized)	0–6 months follow-up (*n* = 17)	0–3 months follow-up (*n* = 8)	3–6 months follow-up (*n* = 9)
Days of hospitalization, mean (SD)	12.2 (10.7)	10.3 (12.5)	14.0 (9.2)
ICU hospitalization, *n* (%)	3 (17.6%)	1 (12.5%)	2 (22.2%)
At most HFNO, *n* (%)	1 (5.9%)	1 (12.5%)	0 (0.0%)
At most NIV, *n* (%)	1 (5.9%)	0 (0.0%)	1 (11.1%)
Intubation, *n* (%)	1 (5.9%)	0 (0.0%)	1 (11.1%)
ECMO, *n* (%)	0 (0.0%)	0 (0.0%)	0 (0.0%)
Dialysis, *n* (%)	1 (5.9%)	0 (0.0%)	1 (11.1%)
CAPA, *n* (%)	2 (11.8%)	1 (12.5%)	1 (11.1%)
COVID-19 related myocarditis, *n* (%)	0 (0.0%)	0 (0.0%)	0 (0.0%)
COVID-19 related mortality, *n* (%)	2 (11.8%)	1 (12.5%)	1 (11.1%)

Abbreviations: CAPA, COVID-19 associated pulmonary aspergillosis; ECMO, extracorporeal membrane oxygenation; HFNO, high flow nasal oxygen; ICU, intensive care unit; NIV, non-invasive ventilation; PrEP, pre-exposure prophylaxis.

## Discussion

PrEP with tixagevimab-cilgavimab in at risk patients with poor seroconversion following prior vaccination may provide protection against severe COVID-19. However, in our lung transplant cohort, 11.0% of lung transplant recipients developed breakthrough SARS-CoV-2 infection/COVID-19 within the initial 3 months post-PrEP, which increased to 17.4% within 6 months. Notably, 27.6% of the patients with breakthrough infection within the first 3 months required hospitalization, while this number increased to 52.9% for those with breakthrough infection during the subsequent 3 months of follow-up. On the other hand, ICU admissions (3.4%) and COVID-19 related mortality (3.4%) only rarely occurred in COVID-19 patients during the first 3 months post-PrEP, whereas ICU admissions non-significantly increased (11.8%) and the number of COVID-related deaths remained similar (5.9%) in the subsequent 3 months of follow-up. Our study demonstrates that COVID-19 breakthrough infections and associated hospitalizations remained an important problem during the Omicron BA.5 surge, despite PrEP with tixagevimab-cilgavimab. Yet, overall ICU admissions (1% post-PrEP) and COVID-19 related mortality (2% post-PrEP) were very low. The latter finding concurs with the observed low mortality rate in an earlier study of SARS-CoV-2 Omicron-variant breakthrough infections in lung transplant patients without PrEP [1].

Al Jurdi et al. reported on 222 SOT recipients (kidney, lung, liver or multi-visceral) who received tixagevimab-cilgavimab PrEP between December 2021 and April 2022, and 222 vaccine-matched solid organ transplant recipients who did not receive PrEP. Breakthrough SARS-CoV-2 infection occurred in 5% of SOT recipients (11 patients) who received tixagevimab/cilgavimab and in 14% (32 patients) of SOT recipients in the control group (*p* < 0.001). This study included 80 lung transplant recipients, of whom 7.5% (6 patients) developed COVID-19 after PrEP during the 4-month follow-up period, and 72 lung transplant recipients without PrEP, in whom 22.2% (16 patients) developed COVID-19 [13]. Of note, the number of prior vaccines varied considerably in these patients, different to our study group in which all patients were fully vaccinated (i.e., two boosters).

Gottlieb et al. analysed their cohort of 419 lung transplant recipients that received PrEP between February and October 2022, with a median follow-up of 209 days. Of these, 19% (77 patients) developed SARS-CoV-2 breakthrough infection, of which 13% (10 patients) were hospitalized and 0.7% (1 patient) died. Notably there was no difference in severity of COVID-19 was observed with the control group that did not receive PrEP, but this could possibly be explained by the fact that both groups were not matched, and patients receiving PrEP were older, had more severe renal insufficiency, shorter time to transplant and lower SARS-CoV-2 antibody titers. Included patients had antibody titers of less than 260 BAU/mL after full vaccination or were included as per decision of their treating physician. Furthermore, most patients also had received double dose PrEP in most cases [15].

Most recently, Sindu et al. reported a 11.8% SARS-CoV-2 breakthrough infection rate and 20.8% hospitalization rate in 203 lung transplant recipients that had received PrEP between December 2021 and August 2022, in comparison to 16.6% and 43.1% in the control group. COVID-19-related mortality was high in both these propensity-score-matched groups (11.8%) [16].

Rates of SARS-CoV-2 breakthrough infections and hospitalization during the first 3 months of follow-up in our cohort are comparable with the studies performed by Al Jurdi et al. and Sindu et al, which took place in the same timeframe, although we—fortunately—note a lower percentage of COVID-19 related mortality. When comparing our overall results (June 2022 to December 2022, 6-month follow-up) to Gottlieb et al (February 2022 to October 2022, median 6.9-month follow-up), we found a similar SARS-CoV-2 breakthrough infection rate but noted a higher hospitalization rate.

When looking at epidemiological data for Belgium between November 2022 and January 2023, BQ.1, a sub-variant of BA.5, became the most dominant circulating SARS-CoV-2 variant [19, 20]. As stated by the Belgian Health Authorities at the end of November, tixagevimab-cilgavimab was deemed ineffective to neutralize the BQ.1 Omicron variant of SARS-CoV-2, necessitating termination of the PrEP program [20, 21]. Although we have no data available on the actual variants in our patient group, this epidemiologic evolution could explain the difference in hospitalization rate we noted between the first 3 months of follow-up and the subsequent 3 months, and the difference in hospitalizations compared to the Gottlieb et al. cohort [15]. Single dosage instead of double dosage, as well as inclusion of patients with a higher antibody count prior to PrEP may have further added to the difference in hospitalization rate compared to the Gottlieb et al. study. Also, the neutralizing effect of tixagevimab-cilgavimab PrEP for Omicron BA.5 has been reported to wane by 3 months post-injection, which might also have contributed to the surge in moderate to severe disease during the last 3 months of follow-up in our cohort [22].

We note several limitations to our study. First, its retrospective and observational design. Second, the incidence of COVID-19 in our study group may be underestimated as we relied on patients getting tested when experiencing symptoms and reporting back on PCR results when diagnosed outside our hospital. However, centralized documentation of a positive SARS-CoV-2 PCR test allowed cross-checking of breakthrough infections for the current study yet genotyping of SARS-CoV-2 was not systematically performed. Furthermore, our study lacked a control group because almost all patients who were eligible for PrEP agreed to treatment, and COVID-19 infections in patients without PrEP were not prospectively collected for analysis during the study period. However, the clinical demographics of the included patients are overall representative of our total lung transplant cohort (i.e., about 25%–30% of patients with CLAD, 30% with diabetes, 15%–20% with chronic kidney disease stage 4, and 25%–30% with heart disease; and about 90% on a tacrolimus-based immunosuppression). None of the included patients was treated with a m-TOR inhibitor, nor received TLI, rATG or rituximab for progressive CLAD in the weeks prior to PrEP administration (as patients needed to be clinically stable to safely allow PrEP administration).

In conclusion, our results add real-world evidence on COVID-19 breakthrough infections after PrEP with tixagevimab-cilgavimab in fully vaccinated LTx recipients with deficient seroconversion; and demonstrates a similar rate of infection after PrEP during the Omicron BA.5 surge as reported in other studies. However, COVID-19 associated hospitalization remained an important problem, despite PrEP with tixagevimab-cilgavimab, whereas severe COVID-19 necessitating ICU admission and COVID-19 mortality were low. Waning of the neutralizing effects of PrEP and changing circulating SARS-CoV-2 variants might possibly explain the increase in SARS-CoV-2 breakthrough infections and hospitalizations after PrEP, and highlights the need for novel, long-term effective PrEP strategies in these high-risk patients.

## Data Availability

The raw data supporting the conclusion of this article will be made available by the authors, without undue reservation.

## Abbreviations

AR, arrythmia; AZA, azathioprine; BAU, Binding Antibody Units; BMI, body mass index; BOS, bronchiolitis obliterans syndrome; CAPA, COVID-19 associated pulmonary aspergillosis; CLAD, chronic lung allograft syndrome; CKD, chronic kidney disease (defined as pre-existing severe chronic renal insufficiency stage 4, with eGFR <30 mL/min/1.73 m^2^); CS, corticosteroids; CSA, cyclosporine A; DM, diabetes mellitus; FEV_1_, Forced Expiratory Volume in one second; HD, heart disease; HF, non-ischemic heart failure with or without preserved ejection fraction; HFNO, high flow nasal oxygen; ICU, intensive care unit; IHD, ischemic heart disease; IQR, interquartile range; LTx, lung transplant; MMF, mycophenolate mofetil; MV, mechanical ventilation; NIV, non-invasive ventilation; PrEP, pre-exposure prophylaxis; RAS, restrictive allograft syndrome; SARS-CoV-2, severe acute respiratory syndrome coronavirus 2; SD, standard deviation; SOT, solid organ transplant; TAC, tacrolimus; UK, unknown.
